# Plant-Based Diet and Sports Performance

**DOI:** 10.1021/acsomega.4c07560

**Published:** 2024-11-07

**Authors:** Tatiana Cantarella Sarmento, Rosângela dos Santos Ferreira, Octávio Luiz Franco

**Affiliations:** † S-Inova Biotech Postgraduate in Biotechnology, Catholic University Dom Bosco (UCDB), Campo Grande 79117-900, Brazil; ‡ Center for Proteomic and Biochemical Analysis, Postgraduate Program in Genomic Sciences and Biotechnology, Catholic University of Brasilia (UCB), Brasilia 70990-160, Brazil

## Abstract

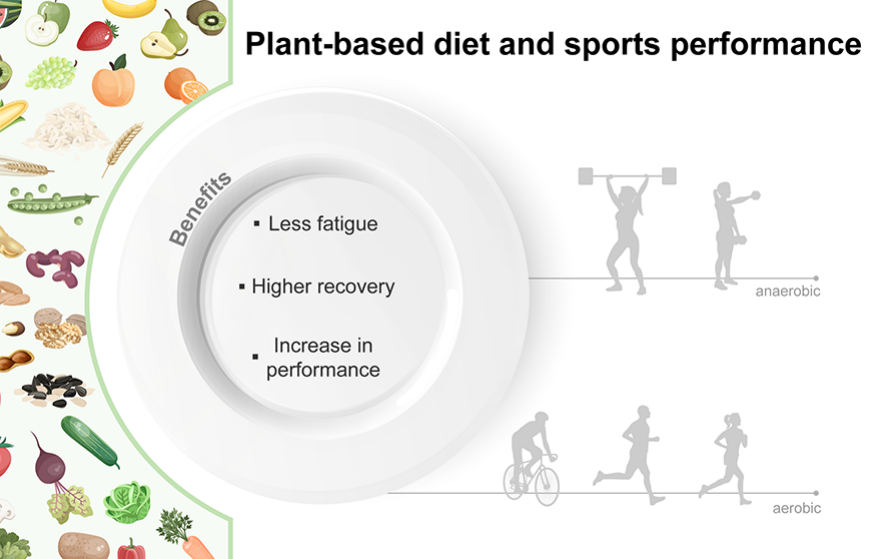

Recently, interest in plant-based diets has grown significantly, driven by health and environmental concerns. Plant-based diets offer potential health benefits, including decreased risk of cardiovascular disease, weight management, and blood glucose regulation. This diet profile is rich in complex carbohydrates, antioxidants, dietary fiber, and phytochemicals. However, antinutrients in some plant foods can make nutrient absorption difficult, necessitating careful dietary planning. Plant-based diets can also improve sports performance; in addition, they can positively influence the intestinal microbial community, which can promote health and performance. The present study covered a review from 1986 to 2024 and involved an experimental design with human participants. The main objective was to evaluate the impact of plant-based diets on sports performance. Recent research suggests that plant-based diets do not harm athletic performance and may positively impact sports performance by improving blood flow and reducing oxidative stress. These findings have potential clinical significance, particularly for athletes seeking to optimize their physical capabilities through dietary interventions

## 1 Introduction

It is estimated that of the number of existing vegetarians in the world, only 75 million are vegetarian by choice, and 1.425 billion are vegetarian by necessity. This estimate considers that those who do not consume meat because they cannot afford it would probably do so if their situation changed.^1^ Despite this, the number of people interested in reducing their consumption of animal products and increasing their consumption of vegetables is growing.^2^

Aside from potential health and ecological advantages, adopting a plant-based diet can yield performance-boosting effects for the body.^3^ This is attributed to increased soluble and nonsoluble dietary fiber intake, vitamin E, vitamin K, beta-carotene, and manganese.^4^ However, certain plant-based foods also encompass antinutrients like phytates and tannins, which can diminish the absorption of vital nutrients.^5^ However, the benefits that a plant-based diet can provide are remarkable, ameliorating lipid levels in the bloodstream,^6^ regulating blood pressure, managing body weight,^7^ and overseeing blood glucose levels.^8^ Furthermore, in individuals with coronary artery disease, a vegetarian diet may be considered appealing to reduce C-reactive protein, a marker of cardiovascular risk.^9^

The use of plant-based whole foods can provide the greatest benefits, as it substantially increases the consumption of fibers, vitamins, minerals, and phytochemicals, in addition to positively modulating the intestinal microbiota and reducing the consumption of all the harmful elements, such as animal protein and saturated fat, in comparison to omnivorous diets.^10^ The vegan diet has been favorably connected to reducing cardiovascular disease risk markers such as reduced body mass index values, total serum cholesterol, serum glucose, inflammation, and blood pressure.^11^

This type of diet can also contribute to improving performance and recovery in sports activities, such as endurance^12,13^ (e.g. running, bike, triathlon) and muscle strength^14−16^ (e.g., weight lifting, CrossFit). Many athletes have dietary patterns deficient in carbohydrates, reaching less than 46% of the daily recommendation,^17^ risking a rapid depletion of liver and muscle glycogen and leading to early muscle fatigue.^18^ In summary, this Review aims to shed some light on the pros and cons of consuming an exclusively plant-based diet for athletes, recommending the fundamental importance of nutritional analysis in the structuring and preparation of a food plan according to individual needs and supplementing nutrients when necessary.

The current Review summarizes the current state of research concerning the implications of a plant-based diet for health and exercise performance in humans, covering articles from 1986 to 2024. The bibliographic search was conducted in PubMed, using the following keywords: “vegetarian diet”; “performance”; “microbiota”; “endurance”; “muscle strength”; and “oxidative stress.” The search strategy was (“vegetarian diet”) AND (“performance” OR “microbiota” OR “endurance” OR “muscle strength” OR “oxidative stress”).

## 2 Plant-Based Food Patterns

Plant-based diets conceptually are vegetarian and vegan diets, where the vegetarian diet eating pattern consists of different types or categories^19,20^ (Table 1). These are the lacto, ovo, or lacto-ovo vegetarian diets that abstain from meat, poultry, and fish but incorporate dairy, eggs, or both.^21^ Less restrictive diets, such as flexitarian or semivegetarian, additionally include low to moderate amounts of red meat, poultry, fish, and seafood, usually including red meat no more than once a week.^22^ There is also the pescatarian diet that eliminates meat and poultry but recommends the consumption of fish and seafood.^23^ The vegan diet is restrictive and considers environmental sustainability, in addition to the philosophical condition. The vegan dietary pattern consists of foods based on whole grains, soy, legumes, nuts, seeds, vegetables, fruits, water-soluble extracts of chickpeas, soybeans, oats, rice, quinoa, amaranth, almonds, cashews, hazelnuts, walnuts, coconut, sesame, and sunflower.^24^ All types of products of animal origin—food, clothing, and other purposes—are restricted. However, it is important to highlight that the quality of foods included in vegetarian and vegan diets must maintain the status of healthy foods, since ultraprocessed food products, such as vegetable sausages and burgers, may contain high concentrations of salt and saturated fat.^25^

**Table 1:** Plant-Based Dietary Pattern

categories	definition	food groups
veganism	a philosophy and way of living that excludes all forms of animal exploitation for food, clothing, or any other purpose^20^	fruits, vegetables, seeds, oil seeds, nuts, grains, roots, and plant-based beverages
ovolactovegetarianism	omits all animal-based foods except eggs, milk, and dairy products^21^	fruits, vegetables, seeds, oil seeds, nuts, grains, roots, plant-based beverages, milk, eggs, and dairy
flexitarian/semivegetarian	includes low to moderate amounts of red meat, poultry, fish, and seafood, usually including red meat no more than once a week^22^	fruits, vegetables, seeds, oil seeds, nuts, grains, roots, plant-based beverages, red meat, poultry, fish, and seafood
pescetarianism	excludes food of animal origin, except fish and seafood^23^	fruits, vegetables, seeds, oil seeds, nuts, grains, roots, plant-based beverages, fish, and seafood

In view of this, the standardization of the nomenclature of vegetarian diets must be objective, as it is essential to correctly define the dietary intervention and its effects in *in vivo* studies, thus avoiding confusing and wrong interpretations. In the case of studies on the performance of recreational or professional athletes, it is valuable to convey the type or category of vegetarian diet, since, in the face of dietary restrictions of animal origin, the corresponding nutritional deficiencies are identified, well understood, and therefore supplemented.^26^

In the case of a vegan diet, it is essential that it is properly designed, containing a variety of plant foods in the routine diet, in addition to satisfying energy and macro- and micronutrient needs. Fortified foods and/or nutritional supplements are introduced in appropriate quantities when convenient.^27,28^

The dietary pattern of vegetarian and vegan diets can improve metabolic health, exhibiting better serum lipid levels and blood sugar control in the general population. In addition to being able to influence the increase in the maximum oxygen volume (VO^2^ max) of recreational and professional athletes, it can have a positive impact on physical and sporting performance, adaptation, and recovery.^29,30^ Studies indicate better indicators of nutritional status, such as decreased fat mass, reduced inflammation, and oxidative stress resulting from exercise. Above all, there are increased levels of some nutrients, such as complex carbohydrates, and, consequently, increased glycogen availability in athletes.^31,32^

The nutritional quality status of vegetarian and vegan diets, due to the chemical composition and, in particular, the high concentration of antioxidant and phytochemical substances, is directly related to health benefits, preventing or assisting in the treatment of chronic diseases. In addition, they can provide positive effects on performance in various types of physical activity.^5^

## 3 Plant-Based Diet: Benefits and Risks

The plant-based diet was created in 1980 by Dr. Thomas Collin Campbell to distinguish a healthy vegetarian diet from an unhealthy one (with refined and processed foods).^33^ Originally, a plant-based diet consisted of whole foods, seeds, vegetable oils, minimally processed foods, spices, fruits, and vegetables, completely excluding animal foods, such as beef, fish, pork, eggs, honey, and dairy products.^34^ As a complement, this kind of food also includes mushrooms and algae.^35^ Although the term plant-based diet was proposed to be synonymous with a restricted healthy diet, the food industry has incorporated this term and defined it as ´́food made from plants that does not contain animal derivatives̀̀.^36^ In this way, it is possible for industrialized and ultraprocessed foods to add inappropriate substances, such as hydrogenated fats, sugars, dyes, or food additives, to enhance flavor, for example. It also makes it possible to exclude many nutrient substances, such as fiber, vitamins, minerals, and phytochemicals, thus contradicting the essence of the term healthy eating in the principles of a plant-based diet.^2^

**Figure 1:**
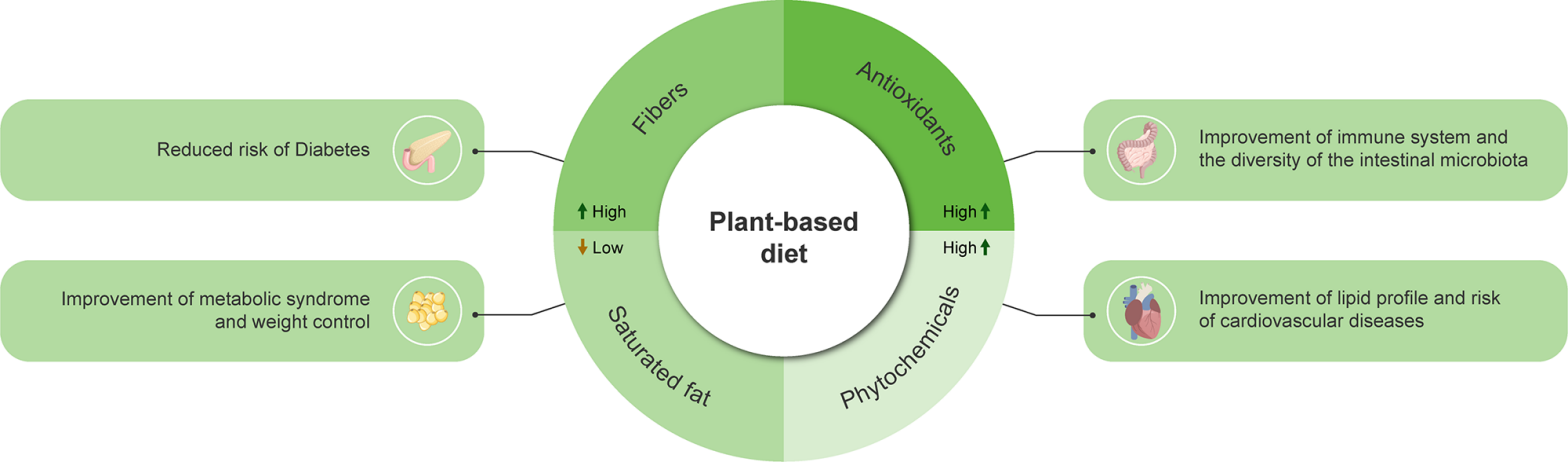
Benefits of the plant-based diet. The up arrow means an increase in fiber, antioxidants, and phytochemicals, and the down arrow means that a reduction in the consumption of saturated fats indicates a reduction in the risk of metabolic diseases, such as diabetes mellitus and obesity, improving the metabolic syndrome. High levels of antioxidants positively impact the immune system, the diversity of the intestinal microbiota, and phytochemicals, having a positive effect on the lipid profile and helping to reduce the risk of cardiovascular disease.

Observational data highlight that vegetarian diets improve cardiovascular risk indices for consumers, reducing morbidity and mortality from ischemic diseases,^37^ type 2 diabetes,^38^ and metabolic syndrome^39,40^ when compared to those who consume omnivorous diets. Further research indicates that vegetarian and vegan diets yield favorable alterations in the lipid profile, significantly reducing plasma cholesterol, low-density lipoprotein, and triglyceride levels.^7,8^ Furthermore, these diets exert a discernible influence on weight management and the reduction of body fat content,^41^ mentioned in Figure 1.

A study undertook a randomized intervention involving 244 individuals with a body mass index ranging between 28 and 40 kg·m^–1^. The participants were allocated to either a low-fat plant-based diet group (*n* = 122) or a control group consuming an omnivorous diet (*n* = 122), spanning a 16-week duration. Notably, alongside evaluating body composition via dual-energy X-ray absorption, the thermic effect of food was quantified, and 44 participants underwent quantification of hepatocellular and intramyocellular lipids through proton magnetic resonance spectroscopy. The intervention group demonstrated substantial weight loss (−5.9 kg) coupled with reductions in insulin resistance (−1.3 HOMA-IR units), hepatocellular lipids (−34.4%), and intramyocellular lipids (−10.4%). A noteworthy 14.1% increase in the thermodynamic effect of food was observed within the intervention group. Conversely, the control group exhibited no alterations in any parameter. The researchers concluded that the adoption of a low-fat, plant-based diet engenders weight loss via diminished energy intake and heightened postprandial metabolism. These shifts were concomitantly linked to reductions in hepatocellular and intramyocellular fat, as well as augmented insulin sensitivity.^42^ More research is needed to confirm whether the favorable outcomes obtained in the study were due to the plant-based diet or the low-fat diet.

Turning to the realm of antioxidant compounds, an investigation encompassed over 3100 globally consumed foods, beverages, spices, herbs, and supplements.^43^ The findings highlighted an average antioxidant content of 0.18 mmol/100 g in animal-derived foods juxtaposed with 11.57 mmol/100 g in plant-derived foods. In essence, foods of plant origin (fruits, vegetables, and nuts) are 5–33 times richer in antioxidants than animal products. Plant-based diets are therefore an excellent source of these nutrients.^43^

Phytochemicals affecting health are remarkable in plants, as they contain many bioactive compounds.^44^ These compounds have the ability to influence various body systems, modulate anti-inflammatory action and nitric oxide production,^45^ as well as influence the control of pathogens from viral infections.^46,47^ Phytosterols, lipid compounds (steroids) derived from plants, represent the largest unsaponifiable lipid fraction in plants. Most phytosterols are unrefined plant oils, present in oleaginous foods (sesame, sunflower, soy, macadamia, almond, and olive), whole grains, and legumes. Their best-known representatives are beta-sitosterol, campesterol, and stigmasterol.^48^ Phytosterols modulate inflammatory and antioxidant responses and have antiulcer, immunomodulatory, antibacterial, and antifungal activities, in addition to their recognized cardiovascular effects due to their ability to inhibit platelet aggregation and reduce total cholesterol and low-density lipoprotein (LDL) levels by 7–12.5% in a dose of 1.5–3 g/day.^48^ Moreover, a well-planned vegetarian diet, composed of natural and whole foods, contains a considerable amount of fiber.^2^ The viscosity of fibers, especially the soluble ones, delays small intestine peristalsis, providing more satiety, favors the slower absorption of nutrients, reducing the glycemic index of the food,^49^ and also confers the formation of more voluminous and softer feces.^9^ Fiber’s effects on glycemic control are well-known, demonstrated in a meta-analysis with improvements in insulin sensitivity, glycated hemoglobin, lipid profile, body weight, and C-reactive protein level. Based on these findings, increasing daily fiber intake by 15 or 35 g may reduce the risk of premature mortality in adults with diabetes.^50^ Due to its ability to bind to various intestinal compounds, fiber increases fecal excretion of cholesterol and bile salts, in addition to providing a substrate for bacterial fermentation, thus modulating the intestinal microbiota and generating several compounds beneficial to metabolism.^51^ Studies suggested that fibers have extra intestinal effects linked to a possible reduction in the activity of 3-hydroxy-3-methyl-glutaryl-CoA reductase, a key enzyme in cholesterol synthesis, in addition to modulating LDL receptors, cholesterol 7 alpha-hydroxylase and mitogen-activated protein kinase, and other genes related to lipid metabolism.^9^ A meta-analysis showed that for every 10 g increase in fiber intake per day, the risk of cardiovascular disease was reduced by 9%, that of coronary disease was reduced by 11%, and the risk of all cancers was reduced by 6%.^52^ The effect of fibers on the prevention of colorectal cancer (adenoma) is also evidenced, both in prevalence and in incidence, and seems to be more closely associated with the protection of men than women.^53^

It is essential to highlight nutritional guidance for adopting a diet excluding products of animal origin, which can cause deficiencies in several nutrients, vitamin B12, folate, zinc, calcium, and iodine.^54,55^ Despite this, studies indicate that vegetarians have good long-term health and may even be better than nonvegetarians when it comes to obesity, heart disease, and some types of cancer. More research is needed to clarify these points and especially the long-term health of vegetarians. Another study analyzing vitamin B12 and folate concentrations in British men following omnivorous, vegetarian, and vegan diets^55^ shows that vegans had lower concentrations of vitamin B12, but not folate, compared to the other groups. In this same study, the use of vitamin B12 supplements was more frequent in vegetarians and vegans. Therefore, more research is needed to evaluate the long-term impact of vegetarian and vegan diets on health, with a focus on potential nutritional deficiencies and their effects.

Another advantage of the plant-based diet is that this kind of diet involves ingesting raw or minimally cooked plant-based foods, characterized by their intact cellular structures, which offers a greater amount of material for use by intestinal microbiota.^56^ Conversely, an ultraprocessed dietary regimen undergoes swift absorption within the small intestine, subsequently robbing the colon of essential nutrients and inducing shifts in both the composition and metabolism of the intestinal microbiota.^57^ A significant influence on a healthy diet is the amount and quality of fat, as it affects the composition of the intestinal microbiota, since a plant-based, low-fat diet increases the population of *Bifidobacteria*. Poly- and monounsaturated fats increase the *Bacteroidetes*:*Firmicutes* ratio, as well as the population of lactic acid-producing bacteria, *Bifidobacteria* and *Akkermansia muciniphila*.^58^ The consumption of nuts, oleaginous food, can increase *Ruminococceae* and *Bifidobacteria*, as well as reducing *Clostridium* sp.^57^ However, adopting an adequate plant-based diet has benefits for the intestinal microbiota, optimizing strain diversity, reducing the most pathogenic bacteria, reducing inflammation levels, and producing more short-chain fatty acids (SCFAs),^59,60^ due to the gut microbes *Roseburia*, *Eubacterium retale*, and *Ruminococcus bromii*, which are found in large amounts in those who consume more complex carbohydrates.^61^ SCFAs are substrates for maintaining colonocyte health, and they contribute to maintaining the intestinal barrier and preventing endotoxemia and its secondary inflammatory effects.^62^ SCFAs have a protective role in type 2 diabetes,^63^ inflammatory bowel disease, and autoimmune diseases;^64^ they also promote immunity against pathogens and are important for microglial function and for the maturation and control of blood–brain barrier integrity.^65^ Still in this context, vegetarian diets have a good cardiovascular metabolic profile, represented by increased production of SCFAs and reduced production of trimethylamine *n*-oxide and secondary bile acids, as they lead to the increase of some bacterial strains (*Prevotella*, *Candida albicans*, *Faecalibacterium prausnitzii*, *Clostridium cluster*, *Roseburia*, *Ruminococcus*, and *Parabacteroides distasonis*), while reducing others, such as *Bilophila wadsworthia*, *Alistipes putredinis*, and *Escherichia coli*.^66^

Nutritional deficiencies are possible, such as vitamin B12 deficiency. This vitamin is present mainly in products of animal origin, so it may be lacking in vegans, and deficiency can cause anemia and neurological problems. Deficiencies in other nutrients such as selenium, zinc, niacin, vitamin B2, vitamin B6, and calcium may also occur, and supplementation may be necessary.^67^ Regarding bone health, studies indicate that vegans may have lower bone mineral density and a higher risk of fractures compared to people who consume meat. Adequate intake of calcium (i.e., 800 mg/d) and vitamin D (i.e., 600 IU/day) is needed, thus indicating that these supplementations may be necessary and crucial to minimize this risk.^68^ The total creatine concentration in skeletal muscle tissue differs between vegetarians and omnivores, with omnivores exhibiting higher concentrations.^69^ This means that omnivores’ muscles can store more creatine, a substance important for energy production during high-intensity exercise.^70^ Creatine is naturally found in animal-based foods, such as meat and fish, and omnivores generally consume more of these foods than vegetarians.^71^ Furthermore, the diminished intake and reduced concentrations of creatine 35–39% lower in blood and 50% lower in muscle, attributed to plant-based diets in comparison to omnivorous diets, can potentially impede sports performance.^22^

Therefore, considering the possible nutritional deficiencies of a plant-based diet, it is essential to develop a specific and individualized dietary plan, with clinical and laboratory monitoring being of the utmost importance. This periodic control may indicate the replanning of the daily diet with higher concentrations of nutrients, fortified foods, and nutritional supplements, when signs of insufficiency begin.

## 4 Plant-Based Diet and Sports Performance

Throughout history, animal-derived protein has been regarded as a fundamental constituent of athletes’ dietary regimens, prompting some researchers to scrutinize the sufficiency and nutritional requisites of vegetarians and vegans in relation to supporting athletic performance.^3^ In historical contexts such as the ancient Olympics, a prevailing recommendation involved the consumption of substantial quantities of meat to attain physical strength.^72^ Ancient narratives from Milon of Crotona, an accomplished Greek wrestler who achieved victory at the Olympic Games on six occasions, reported him consuming 9 kg of meat, 9 kg of bread, and 8.5 L of wine to prepare for his contests.^72^ However, based on scientific evidence, a plant-based dietary approach appears to underpin athletic performance while simultaneously contributing to physical well-being and environmental sustainability.^3^

Physical exercise triggers an acute inflammatory response characterized by an increase in specific cytokines (such as IL-1β, TNF-α, and IL-6), acute phase proteins (such as CRP), hormones, cell adhesion molecules, and the activation of polymorphonuclear leukocyte (PMNL). This inflammatory response generally aids muscle recovery.^73^ In addition, predominantly plant-based diets provide many bioactive compounds with antioxidant properties, capable of mitigating oxidative stress induced by physical exercise and favoring muscle tissue repair. However, an imbalance between training and rest can lead to chronic inflammation, impairing performance.^74^ Diet choice is crucial in sports performance and recovery,^75^ and the plant-based diet has gained prominence due to its benefits for overall health. In the meantime, the literature still lacks in-depth studies of the specific impacts of this diet on the immune response during exercise. A systematic review showed an association between the vegan diet and lower CRP levels compared to those of omnivores. This association was less pronounced in vegetarians, and no substantial effect was observed for all other inflammatory biomarkers, such as IL-6 and TNF-α.^76^ Studies on immune system activation refer to foods of plant origin with functional components such as phenolic, anthocyanins, flavonoids, catechins, and others, forming and producing immune system cells (T and B cells). In addition to food sources of minerals, such as zinc, magnesium, selenium, copper, and iron, they contain and stimulate the production of selenoproteins and neutrophils. PBD also includes a high content of vitamins, such as A, C, D, E, B6, and B9, which protect and form antioxidant substances and antibodies.^77^ All of these plant bioactives enhance immunomodulatory activities and generate performance.

**Figure 2:**
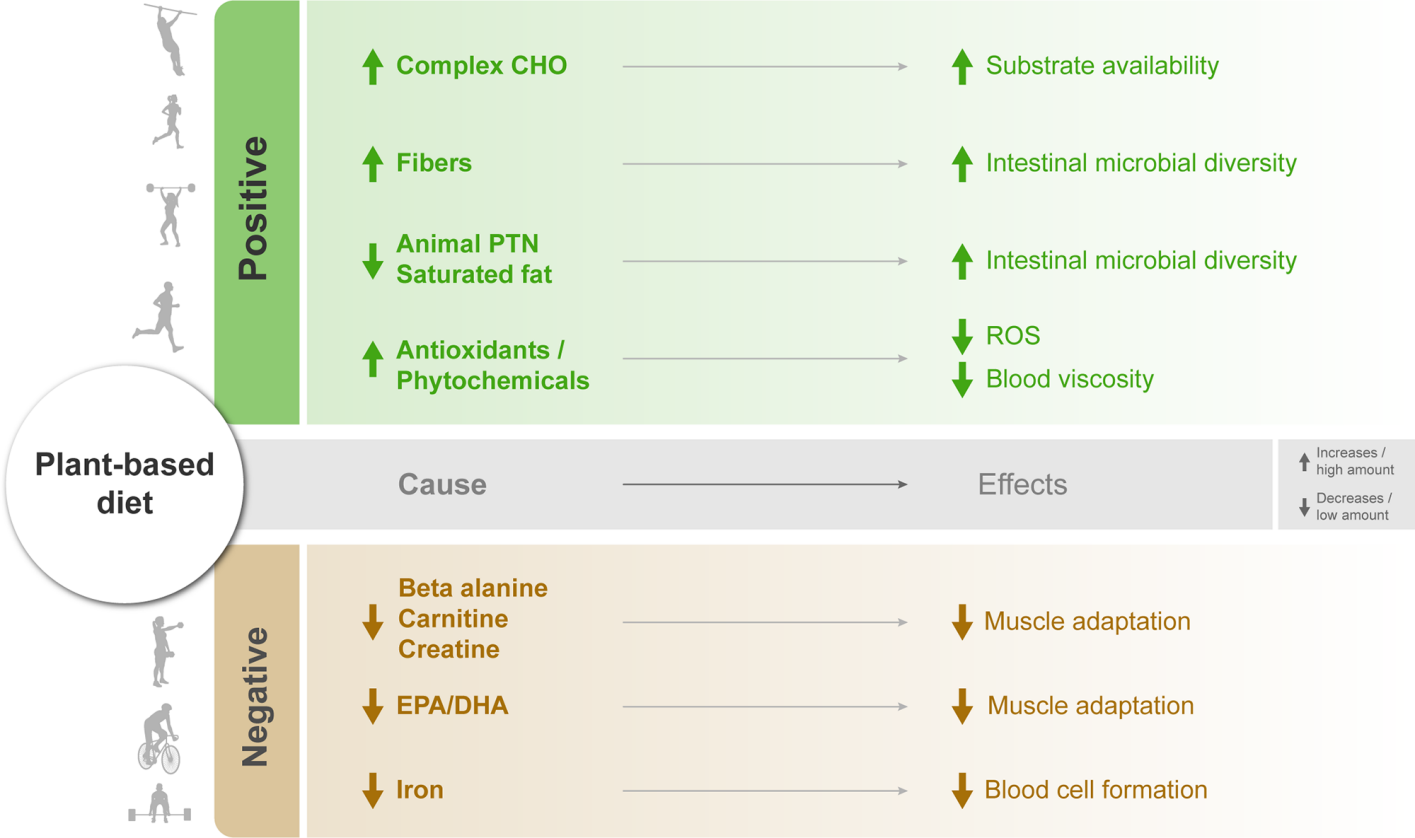
Impact of dietary properties of a plant-based diet on physiological subsystems and sports performance. Dietary factors that have a positive effect (green color) on sports performance are high amounts of carbohydrates (CHO), fiber, antioxidants, and phytochemicals due to the increased availability of energy substrates, increased diversity of the intestinal microbiota, and the reduction of reactive oxygen species (ROS) and blood viscosity, respectively. Factors that cause adverse effects (yellow color) on sports performance are that deficits of beta-alanine, carnitine, creatine, eicosapentaenoic acid (EPA), and docosahexaenoic acid (DHA) lead to reduced muscle adaptation, and low iron intake leads to reduced blood cell formation.

As shown in Figure 2, some impacts from the dietary properties of the plant-based diet on physiological subsystems and sports performance will be reported. The vegans consumed less saturated fat and significantly more linoleic acid than the omnivores. The vegan diets are devoid of arachidonic acid, eicosapentaenoic acid (EPA), and docosahexaenoic acid (DHA).^78^ However, some investigations in humans suggest that the supplementation of EPA and DHA may yield increased muscle mass and strength,^79^ potentially stimulating mTOR-related signaling and protein synthesis.^80^ Conversely, one study over an 8-week period did not observe a significant increase in response to an acute resistance exercise session compared to a placebo group with coconut oil as a control.^81^

A pivotal advantage of a plant-based diet lies in its elevated content of complex carbohydrates, predominantly in the form of polysaccharide molecules found in hepatic and muscle cells. These molecules serve as energy reserves, facilitating optimal liver and muscle glycogen loading.^32^ Another advantage under exercise is blood volume increase from aerobic training, with the associated expansion of plasma volume outpacing the growth of red cell mass, thereby potentially mitigating blood viscosity.^82^ Dietary choices exert an influence on the plasma viscosity as well. Given the typically low saturated fat content and absence of cholesterol in plant-based diets, vegetarian dietary patterns manifest a reduction in plasma lipid concentrations, consequently contributing to lowered viscosity.^27^ One study comparing 48 individuals following a vegetarian diet, with 41 omnivorous individuals as a control group, observed that levels of blood viscosity, a key element to carry oxygen to other tissues, and cell volume were lower in the vegetarian diet group when compared to omnivores.^83^ Thus, a plant-based diet may contribute to blood viscosity reduction, improving blood flow, which is associated with sports performance.^84^ In another intervention study with men and women, moderately trained CrossFit participants demonstrated the positive effect on strength resistance in classic deadlifts on a vegan diet, performed for 4 weeks, with high-intensity functional training.^16^

In addition, the plant-based diet may provide more antioxidants and minerals in food, promoting increased sports performance and health benefits.^3^ On the other hand, as already mentioned, some plant-based foods may contain antinutritional factors, which reduce the bioavailability of essential nutrients for sports.^5^ However, we must also take into account that antinutrient factors such as lectins, oxalates, phytates, phytoestrogens, and tannins may have positive effects on human health.^6^ Furthermore, cooking methods such as soaking, sprouting, fermenting, and cooking can reduce these antinutritional factors.^85^ The main elements considered to be antinutrients and their clinical implications are listed in Table 2.

**Table 2:** Antinutrients and Clinical Implications

antinutrient	food source	harmful effect	method to minimize antinutrients
lectins^6^	vegetables, cereal grains, seeds, nuts, fruits, and vegetables	changes in gut function and inflammation; have antioxidative, antimicrobial, insecticidal, and antitumor properties; modulate glycemic levels in the blood; inhibit bacterial biofilm formation	soaking, sprouting, cooking, and simmering
oxalates^6,90,91^	herbal teas (leaves, stems, roots), spinach, sweet chard, sorrel, beet greens, beetroot, rhubarb, nuts, legumes, cereal grains, sweet potato, and potatoes	may inhibit calcium absorption and increase the formation of kidney stones	soaking, dehulling, blending, whisking and deep-frying, grinding, cooking, dry roasting or heating or drying, boiling
saponins^91,92^	herbal teas (leaves, stems, roots)	interferes with the absorption of lipids and vitamins A and E	soaking, grinding, cooking, dry roasting or heating or drying, boiling
phytotes^6,87^	legumes, cereal grains, pseudocereals (amaranth, quinoa, millet), nuts, seeds, green peas, and chickpeas	may inhibit the absorption of iron, zinc, and calcium	soaking, sprouting, cooking, and simmering
		has antioxidant and antineoplastic effects	
steroids^93–95^	herbal teas	increased risk of cardiovascular events	soaking, grinding, cooking, dry roasting or heating or drying, boiling
phenolics^91,93^	herbal teas	interferes with decreasing appetite and the bioavailability of amino acids; risk of respiratory and cardiac complications	soaking, grinding, cooking, dry roasting or heating or drying, boiling
goitrogens^6,95^	*Brassica* vegetables (kale, Brussels sprouts, cabbage, turnip greens, Chinese cabbage, and broccoli), millet, goitrogens, and cassava.	hypothyroidism or goiter	soaking, boiling, sieving, coagulating, and dewatering under pressure
		inhibition of iodine uptake by the thyroid	
terpenoids^96^	herbal teas	affects carbohydrate metabolism and can cause hepatoxicity and renal alterations	soaking, grinding, cooking, dry roasting or heating or drying, boiling
phytoestrogens^6^	soy, soy products, and flaxseed	endocrine disruptor	heating it with the evaporation of surface vapor and collection of skin on the top of the milk
		increased risk of estrogen-sensitive cancer	
tannins^6,97^	tea, cocoa, grapes, berries, apples, stone fruits, nuts, beans, and whole grains	inhibition of iron absorption; exhibits antinutritional effect, enhanced indigestibility, mutagenic, carcinogenic, and hepatotoxic activities, negative impact on iron stores	soaking, boiling, and fermentation
		reduction in essential amino acids due to changes in protein digestibility	
alkaloids^6,97^	herbal teas	involved in neurotoxicity	soaking, grinding, cooking, dry roasting or heating or drying, boiling
flavonoids^6,98^	herbal teas	reduce absorption of iron and zinc	soaking, grinding, cooking, dry roasting or heating or drying, boiling
		may interfere with the secretion of digestive enzymes	

It is worth mentioning a cross-sectional investigation aimed at comparing the micronutrient status of 74 recreational runners categorized into the three dietary groups of omnivores, lacto-ovo vegetarians, and vegans. This study showed no vitamin B12 deficiency in any group, with supplement users having higher levels. Folate levels in red blood cells were above the recommended ranges for all participants. Vitamin D levels were similar across all groups with a low prevalence (less than 20%) of deficiency in each group. While less than 30% of participants in each group had low iron stores, none exhibited iron deficiency.^86^ These results suggest that recreational athletes who follow vegetarian or vegan diets may have an adequate status of several micronutrients that are important for performance and health. However, the study evaluated amateur athletes, and the impact of vegetarian/vegan diets may be different in professional athletes. Therefore, it is important that longitudinal studies in the recreational athlete population be conducted to evaluate possible long-term nutritional deficiencies.

Physical exercise, along with a specific diet for athletes and factors involved in training performance, can influence the intestinal microbiota, including bacterial species abundance, microbiota diversity, production of metabolites, improvement in the intestinal barrier function, and enhancement in intestinal mucosa immunity.^87^ Intestinal stress during exercise includes biochemical responses in an effort to recover homeostasis, such as increased permeability, release of cytokines, modification of the microbiota, activation of the hypothalamic-pituitary-adrenal axis, immune modulation, and intestinal hormonal variations.^88^ It is predicted that stress prevalence is higher in endurance sports such as swimming, rowing, skiing, cycling, triathlon, and long-distance running.^89^

Several physiological alterations can occur in athletes who practice endurance sports, since high-intensity exercises and prolonged training can lead to the development of inflammatory bowel diseases, increased permeability of the epithelial wall, and rupture of the intestinal mucosa thickness.^99^ It has recently been demonstrated that 60 min of vigorous resistance training at 70% of maximal work capacity leads to characteristic leaky intestinal responses, which occur primarily due to splanchnic hypo perfusion and subsequent ischemia, impairing nutrient absorption and causing damage to splanchnic cells in the intestinal mucosa.^100^ Therefore, it is common for some athletes to report symptoms such as abdominal distention, acid reflux (heartburn), nausea, vomiting, diarrhea, and cramps, among others. Furthermore, another important factor in sports performance is the impairment of mucosal immunity, which is a risk factor for the emergence of upper respiratory tract infections with a higher incidence in elite athletes.^89^ All this negatively affects the training of athletes and consequently impairs performance during competitions.^79^ Another highlight is that when compared to ordinary individuals, athletes have a greater diversity in the microbiota.^101^ The variety of bacterial phyla is also a differential in athletes; in nonathletes the *Firmicutes* and *Bacteroidetes* phyla are prevalent.^77^ The protein intake could be correlated with an enhanced intestinal microbiota diversity.^102^

A recent investigation conducted a comparative analysis of the cardiovascular fitness levels of two groups: 9 healthy young men adhering to a vegan dietary regimen and 16 individuals following an omnivorous diet. The assessment involved measuring the maximum oxygen consumption (VO^2^ max) achieved on a stationary bicycle. The acquired data did not reveal any discernible discrepancies between the groups in relation to their VO^2^ max values. Even though young, healthy vegan and omnivorous men had very different diets, they showed no significant differences in their blood vessel and skeletal muscle health, function, or cardiovascular fitness.^103^ However, although this study indicates that healthy young people, vegans and omnivores, have similar levels of cardiovascular consumption as measured by VO^2^ max, more research is needed to better understand the long-term effects of vegan diets on athletic performance and overall cardiovascular health. Another study, wherein a plant-based dietary profile was implemented over a span of 14 weeks, exhibited a reduction in body fat, as mentioned in Figure 2, alongside a diminished risk of metabolic ailments. This regimen also yielded an augmented capacity for exercise resistance, coupled with an enhancement in maximal aerobic capacity.^32^ It is worth emphasizing that this particular dietary profile holds the capacity to modulate molecular signaling pathways, involving elements like leucine, creatine, and polyunsaturated fatty acids, all of which are directly associated with adaptations in skeletal muscle functionality.^104^ Current investigations that delve into the comparison between plant-based diets and their influence on athletic performance are comprehensively presented in Table 3.

**Table 3:** Relationship between Plant-Based Diet and Sports Performance^a^

study design	participant	training status	nutritional intervention	exercise intervention	results	authors
Outcomes of training interventions and blood biochemistry parameters. The participants were assigned to follow a vegan diet (VEG) or a traditional mixed diet (Mix).	women (*n* = 12) and men (*n* = 8)	CrossFit participants with moderate training	four-week vegan diet of VEG	participants underwent a one-repetition maximum (1RM) test to determine the maximum weight they could lift once; subsequently, they performed a modified Fight Gone Bad (FGB) test	↑ 1RM in the Mix	Durkalec-Michalski et al. (2022)^16^
					↑ FGB in the Mix	
					→ between groups	
Lean leg mass, total muscle cross-sectional area, and individual muscle fiber cross-sectional area, as well as 1RM leg-press, were evaluated at baseline and following the intervention.	*n* = 38	active physically engaged in resistance training for at least 1 year	usual intake was measured and then modified to achieve 1.6 g kg ^–1^ day ^–1^ via protein supplementation (soy for VEG or whey for OMN)	12-week supervised resistance training (2×/week) leg press 1RM	↑ 1RM in both groups	Hevia-Larraín et al. (2021)^105^
	19 VEG (26 ± 5 years; 72.7 ± 7.1 kg, 22.9 ± 2.3 kg/m^2^)					
	19 omnivorous (OMN) (26 ± 4 years; 73.3 ± 7.8 kg, 23.6 ± 2.3 kg/m^2^)					
Cross-sectional study.	*n* = 70	sports club team		treadmill VO^2^ max test and leg extension peak torque (PTEP) was assessed using a dynamometer for lower extremity strength evaluation	↓ protein intake in VEG	Lynch et al. (2016)^14^
	27 VEG				→ body mass in both groups	
Elite OMN and VEG adult endurance athletes for maximum oxygen uptake (VO^2^ Max) and strength.	43 OMN				↑ VO^2^ max in women VEG	
	age: 21–58 years old				→ VO^2^ max in men of both groups	
					→ PTEP in both groups and genders	
Cross-sectional study.	*n* = 56 young, healthy women	physically active with 150–200 min of cardio per week			↑ VO^2^ max in VEG	Boutros et al. (2020)^107^
Anthropometric measurements, body composition, VO^2^ max, muscle strength (MS), and dietary factors were measured.	age: 25.6 ± 4.1 years					
	28 VEG (for at least 2 years), 28 OMN					
Cross-sectional study.	*n* = 52	physically active, at least 3× a week		PTEP VO^2^ max	↑ VO^2^ max in VEG	Król et al. (2020)^13^
	22 VEG age: 32 ± 5 years				PTEP → between groups	
	30 OMN age: 30 ± 5 years					
Six months of intervention.	group 1 (*n* = 10)		group 1: OMN	VO^2^ max (mL/kg/min) by incremental bike test	→ VO^2^ max and body composition and both groups	Blancquaert et al. (2018)^119^
	age: 25.9 ± 9.0 years					
	group 2 (*n* = 15)		group 2: lacto-ovo vegetarian diet + placebo			
	age: 25.4 ± 7.1 years					
	group 3 (*n* = 14)		group 3: lacto-ovo vegetarian diet + β-alanine and creatine			
	age: 25.5 ± 6.6 years					
Cross-sectional study.	*n* = 25; 16 OMN age: 21 ± 1 years	no history of resistance exercise in the previous six months		VO^2^ max (mL/kg/min) and (L/min) maximum volunteer isometric contraction (MVIC) strength	→ VO^2^ max and MVIC in both groups	Page et al. (2022)^103^
	9 VEG age: 24 ± 3 years					

a Abbreviations: maximum oxygen uptake: VO^2^ max; maximum volunteer isometric contraction: MVIC; modified fight gone bad test: FGB; muscle strength: MS; omnivorous: OMN; peak torque for leg extensions: PTEP; repetition maximum: 1RM; vegan diet: VEG; traditional mixed diet: Mix. Arrows indicate increase (↑), no change (→), or decrease (↓).

Recent research findings highlight that the quality of dietary protein does not differ from the adaptive response to training, a phenomenon intrinsically linked to the composition of the diet itself.^104^ A recent clinical trial carried out by Hevia-Larraín et al. (2021)^105^ revealed that a high-protein diet (∼1.6 g kg^–1^ day^–1^) for young men appears to be unaffected by protein source (plant-based vs animal-based), allowing them to consume enough protein, building muscle strength and mass when following a high-protein diet and resistance training. This condition suggests that research on omnivorous and vegetarian diets, regardless of the modality, should be pursued in order to better clarify the performance of athletes. In a similar vein, a study juxtaposed the cardiorespiratory fitness of two cohorts of 27 elite vegetarian runners.^106^ In it, cardiorespiratory fitness, ascertained through Bruce’s protocol, was quantified in terms of VO^2^ max. The outcomes unveiled a significantly higher relative VO^2^ max in the group adhering to a vegetarian diet among women, though this was not observed among men. In contrast, the absolute VO^2^ max exhibited no noteworthy variance between the two groups. Interestingly, the elevated relative VO^2^ max among vegetarian women was linked to their comparatively lower body mass in comparison to their omnivorous counterparts.^107^ In the study, an increase in relative VO^2^ max was observed only in vegetarian women. It is necessary to investigate whether specific hormonal or metabolic factors influence this result. Furthermore, a correlation between lower body mass index and higher relative VO^2^ max was identified in vegetarian women. However, it has not been determined whether the vegetarian diet directly causes lower body weight.

A recent study compared sprint interval exercise performance between vegans and omnivores. Nine individuals from each group, with similar levels of physical activity, performed four sets of 30 s sprints on a cycle ergometer. Maximum power, average power, fatigue index, and time to reach maximum power were measured. Vegans and omnivores showed similar performances in high-intensity sprints. The vegan diet did not appear to impair performance in exercises that required bursts of strength.^88^ Likewise, a cross-sectional study encompassing 56 physically active young women divulged no significant disparities in muscle strength and body composition between vegan and omnivorous participants. Nevertheless, vegans exhibited a higher estimated VO^2^ max (44.5 ± 5.2 vs 41.6 ± 4.6 mL·kg^–1^) and longer submaximal endurance time to exhaustion (12.2 ± 5.7 vs 8.8 ± 3.0 min) in comparison to their omnivorous counterparts.^108^ In an additional cross-sectional endeavor involving 52 physically active individuals, 22 of whom were vegans and 39 of whom were omnivores, the study’s purview was the influence of a plant-based diet on the performance of amateur athletes and its implications for the morphological and functional remodeling of the heart.^13^ It is worth remembering that vegan diets generally contain less saturated fat^52,66^ and more fiber^9^ and antioxidants,^5,43,109^ which can have great benefits for cardiovascular health.

Turning to the realm of cellular metabolic equilibrium, reactive oxygen species (ROS) emerge as a critical factor (Figure 2). ROS, when present in low or normal concentrations, play a functional role in the body’s defense mechanisms.^110^ Particularly during physical exercises, ROS production escalates, giving rise to oxidative stress. This process, in turn, is linked to the sustenance of abnormal mitochondrial activity and other cellular organelles during exercise, while also influencing cellular responses to tissue damage.^111^ The heightened oxidative stress provoked by intense exercise, particularly at exertion levels beyond 60% VO^2^ max,^112^ can precipitate early fatigue, subsequently impairing sports performance and muscle recovery.^113^ The distinctive dietary profile of plant-based diets can contribute to improved muscle recovery and engender positive effects on exercise outcomes, concurrently augmenting insulin sensitivity.^114^ As underscored earlier in this synthesis, plant-based diets provide elevated levels of dietary fiber, phytochemicals, and antioxidants, which can counteract the effects of ROS generated during exercise, thereby facilitating muscle recovery.^113^

Furthermore, transport of glucose to the pancreas via the type 2 glucose transporter instigates adenosine triphosphate (ATP) production through the Krebs cycle. In the presence of ATP, potassium channels close, while calcium channels open. Functioning as a secondary messenger, ATP prompts the release of insulin into the bloodstream.^115^ However, advanced glycation end products (AGEs) impede ATP production within pancreatic beta cells, consequently restraining insulin release, which is characterized by defects in uptake and oxidation of glucose, a decrease in glycogen synthesis, and, to a lesser extent, the ability to suppress lipid oxidation.^116^ AGEs are nonenzymatically glycated proteins or lipids involving glucose and other reducing sugars, including glyceraldehyde, glycolaldehyde, methylglyoxal, and acetaldehyde.^12^ Glycation can also transpire during cooking processes such as frying, baking, or microwaving, primarily during caramelization.^117^ Notably, advanced glycation is predominant in animal-derived foods due to their elevated protein and fat content.^118^ Controlling blood glucose is essential for health and sports performance. Well-planned vegan diets can help to limit the formation of AGEs and aid glycemic control.

## 5 Final Considerations

The study showed that a plant-based diet can be considered an advantageous option for athletes, as in addition to not negatively influencing sporting results, it can strategically optimize performance and improve health. Monitoring with a specialized nutritionist is of fundamental importance to ensure the success of plant-based nutrition in a sports context. Finally, we suggest that new studies be carried out with an explicit design of the type or category of vegetarian diet used as a dietary intervention. In this way, sporting outcomes will be better understood, in addition to opening paths for innovations in the line of nutritional supplements.

## Data Availability

The data supporting this article have been included as part of the figures and tables. No primary research results, software, or code have been included, and no new data were generated or analyzed as part of this Review.

## Abbreviations

1RM: repetition maximum
AGEs: advanced glycation end products
ATP: adenosine triphosphate
DHA: docosahexaenoic acid
EPA: eicosapentaenoic acid
LDL: low-density lipoproteins
POST: after; PRE before
ROS: reactive oxygen species
SCFAs: short-chain fatty acids
VO^2^ max: maximum oxygen consumption
